# A Matching Strategy To Guide Donor Selection for Ulcerative Colitis in Fecal Microbiota Transplantation: Meta-Analysis and Analytic Hierarchy Process

**DOI:** 10.1128/spectrum.02159-21

**Published:** 2022-12-06

**Authors:** Bangzhou Zhang, Luxi Yang, Hanbing Ning, Man Cao, Zhangran Chen, Qiongyun Chen, Guanghui Lian, Hailing Tang, Qizhi Wang, Junping Wang, Zhihui Lin, Jianbo Wen, Yuedong Liu, Ji Xuan, Xuejun Li, Aiqiang Lin, Jianquan He, Lei Zhang, Xiaohua Hou, Qiang Zeng, Chuanxing Xiao

**Affiliations:** a School of Life Sciences, Xiamen University, Xiamen, China; b School of Medicine, Xiamen University, Xiamen, China; c Department of Digestive Diseases, First Affiliated Hospital of Zhengzhou University, Zhengzhou, China; d Xiamen Treatgut Biotechnology Co., Ltd., Xiamen, China; e Department of Gastroenterology, Xiangya Hospital, Changsha, China; f Department of Gastroenterology, Xi'an Central Hospital, Xi’an, China; g Department of Gastroenterology, The First Affiliated Hospital of Bengbu Medical College, Bengbu, China; h Department of Gastroenterology, The Affiliated People's Hospital of Shanxi Medical University, Taiyuan, Shanxi, China; i Department of Gastroenterology, Fujian Provincial Hospital, Fuzhou, China; j Department of Gastroenterology, Pingxiang People’s Hospital, Pingxiang, China; k Department of Gastroenterology, The Third Affiliated Hospital of Liaoning University of Traditional Chinese Medicine, Shenyang, China; l Department of Gastroenterology, Jinling Hospital, Nanjing, China; m Department of Gastroenterology, The Second Affiliated Hospital of Anhui University of Traditional Chinese Medicine, Hefei, China; n Division of Gastroenterology, Union Hospital, Tongji Medical College, Huazhong University of Technology and Science, Wuhan, China; o Health Management Institute, The Second Medical Center & National Clinical Research Center for Geriatric Diseases, Chinese PLA General Hospital, Beijing, China; p School of Pharmacy, Fujian University of Traditional Chinese Medicine, Fuzhou, China; q Department of Gastroenterology, The Second Affiliated Hospital of Fujian University of Traditional Chinese Medicine, Fuzhou, China; Emory University

**Keywords:** 16S rRNA gene sequencing, meta-analysis, analytic hierarchy process, donor-recipient matching, ulcerative colitis, fecal microbiota transplantation

## Abstract

Fecal microbiota transplantation (FMT) targeting gut microbiota has recently been applied to the treatment of ulcerative colitis (UC). However, preliminary trials showed that only a subset of patients responded to FMT, and the heterogeneity in donor gut microbiota probably played important roles in patients’ responses, implying the significance of matching an appropriate donor to a specified patient. We developed a strategy to build a donor-recipient matching model to guide rational donor selection for UC in FMT. We collected and uniformly reanalyzed 656 fecal 16S rRNA gene sequencing samples (350 from UC patients and 306 from healthy subjects) from 9 studies. Significantly lower α-diversity indexes were observed in UC patients by random effects model. Thirty-four bacterial genera and 34 predicted pathways were identified with significant odds ratios and classification potentials for UC patients. Based on six bacterial indicators, including richness, overall distance, genera, and pathways (beneficial and harmful), the analytic hierarchy process-based donor-recipient matching model was set to rank and select appropriate donors for patients with UC. Finally, the model showed favorable classification powers (>70%) for FMT effectiveness in two previous clinical trials. This study revealed the dysbiosis of fecal bacterial diversity, composition, and predicted pathways of patients with UC by meta-analysis and hereby developed a donor-recipient matching strategy to guide donor selection for UC in FMT. This strategy can also be applied to other diseases associated with gut microbiota.

**IMPORTANCE** Modulation of gut microbiota by FMT from donors has been applied to the treatment of UC and yielded variable effectiveness in clinical trials. One possibility is that this variable effectiveness was related to donor selection, as a patient’s response to FMT may rely on the capability of the used donor’s microbiota to restore the specific gut disturbances of the patient. However, the biggest issues on the practical level are what should be considered in the selection process and how to set up such a donor-recipient matching model. In this study, we presented a bacterial profile-based donor-recipient matching strategy to guide donor selection for UC in FMT by first meta-analysis of 656 fecal 16S rRNA gene sequencing samples from 9 studies to identify significant indicators and then setting up the model by an analytic hierarchy process. The applicability and accuracy of this model were verified in the data sets from two previous FMT clinical studies. Our data indicate that the donor-recipient matching model built in this study enables researchers to rationally select donors for UC patients in FMT clinical practice, although it needs more samples and prospective trials for validation. The strategy adopted in this study to leverage existing data sets to build donor-recipient matching models for precision FMT is feasible for other diseases associated with gut microbiota.

## INTRODUCTION

Ulcerative colitis (UC), one subtype of inflammatory bowel disease (IBD), is a remitting and relapsing inflammatory disease comprising symptoms of abdominal pain, diarrhea, fecal urgency, gastrointestinal bleeding, and weight loss. UC has become a global disease in 21st century, with a high coalescing incidence range of 6 to 15 per 100,000 in the Western world and accelerating incidence in newly industrialized countries (1, 2). Despite the amount of available therapies, including corticosteroids, anti-tumor necrosis factor alpha (TNF-α) agents, aminosalicylates, immunomodulators, and surgery (3), many patients are unresponsive to these treatments or present secondary failure during treatment. Hence, the development of new therapies and the investigation of alternative strategies are needed. While the etiology of UC remains unclear, it is considered to be intricately attributable to genetic, immunological, and environmental factors (4, 5).

Gut microbiota, as an important environmental factor, play important roles in UC progress (6–8). Gut microbiota manipulation by fecal microbiota transplantation (FMT) has demonstrated promising effectiveness in remission of UC as well as for treatment of other diseases in clinical trials (8–12). However, the effectiveness of FMT varied among different studies (13, 14), in which specified donors may play crucial roles. For example, a randomized controlled trial of FMT for UC using 5 donors showed 78% of patients achieved remission after they received stool from a single donor (11). Also, patients who received FMT batches containing stool from the same donor exhibited a higher remission rate (37%) than those whose FMT batches did not include this donor (18%) (9). Although large clinical trials are needed to be prospectively designed to further determine donor-specific effects (15), these published studies imply the importance of donor selection for success of FMT (16). However, the general approach to donor selection in FMT trials is to use a single healthy donor or to randomly select multiple donors from a set of screened potential donors (9). Improved, rational donor selection could potentially reduce the risks of false-negative trials and accelerate the development of novel therapies for microbiome-mediated conditions (17).

Given the variation in donor microbiota and the potential impact on clinical efficacy, how should researchers precisely select a donor for a specified patient? This may be addressed by matching donors and recipients. A recent study reported the importance of age matching between donors and recipients by showing the lower cumulative nonrelapse rate in the group with bigger age difference between the donor and the UC patients (18). Moreover, existing trials support that effectiveness is associated with higher microbial richness (19) and enrichment in members of the *Lachnospiraceae* family and *Ruminococcus* genus in donors (11, 20). Indeed, significant improvement was obtained after FMT from a single highly selected donor based on clinical and microbial criteria (21). More and more researchers are applying donor-recipient matching based on gut microbiota profiles for diseases like UC and IBS, although the specific parameters for implementing a selection critieria are still unknown (22, 23). Fortunately, the rapid expansion in publicly available microbiota sequencing data sets and the emergence of large stool banks (e.g., Openbiome [24]) make it feasible to identify microbial indications, build models, and select donors rationally from a large donor pool during the FMT clinical trials (17).

**FIG 1:**
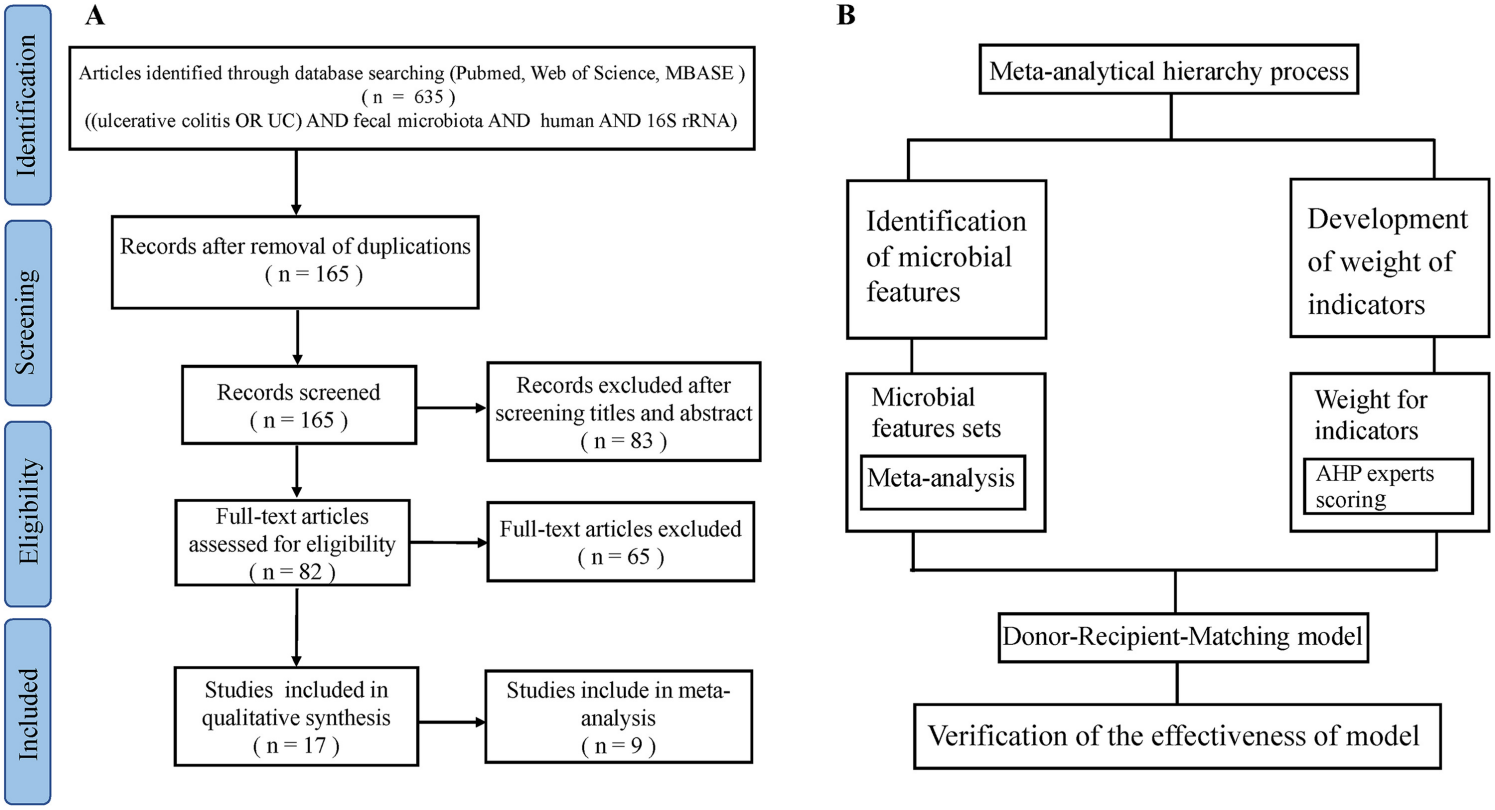
PRISMA flow diagram depicting the search results and selection of included studies in this systematic review (A) and the process for building the donor-recipient matching model by meta-analysis and an analytical hierarchy process (AHP) (B).

In this article, we present pioneer work to guide donor selection for patients with UC in FMT trials by a matching strategy integrating meta-analysis (25) and an analytical hierarchy process (AHP) (Fig. 1). We first collected and uniformly reanalyzed publicly available fecal 16S rRNA gene sequencing data of UC patients and healthy controls (HCs) from 9 studies. By meta-analysis of features with significant odds ratios (ORs) and classification potentials, we sought to determine the shifts of bacterial α-diversity, β-communities, bacterial taxa, and pathways in patients. With these indications, we built a donor-recipient matching model by AHP and tested the applicability and accuracy of this model in two previous UC-FMT clinical trials.

## RESULTS

### Characteristics of included studies.

**FIG 2:**
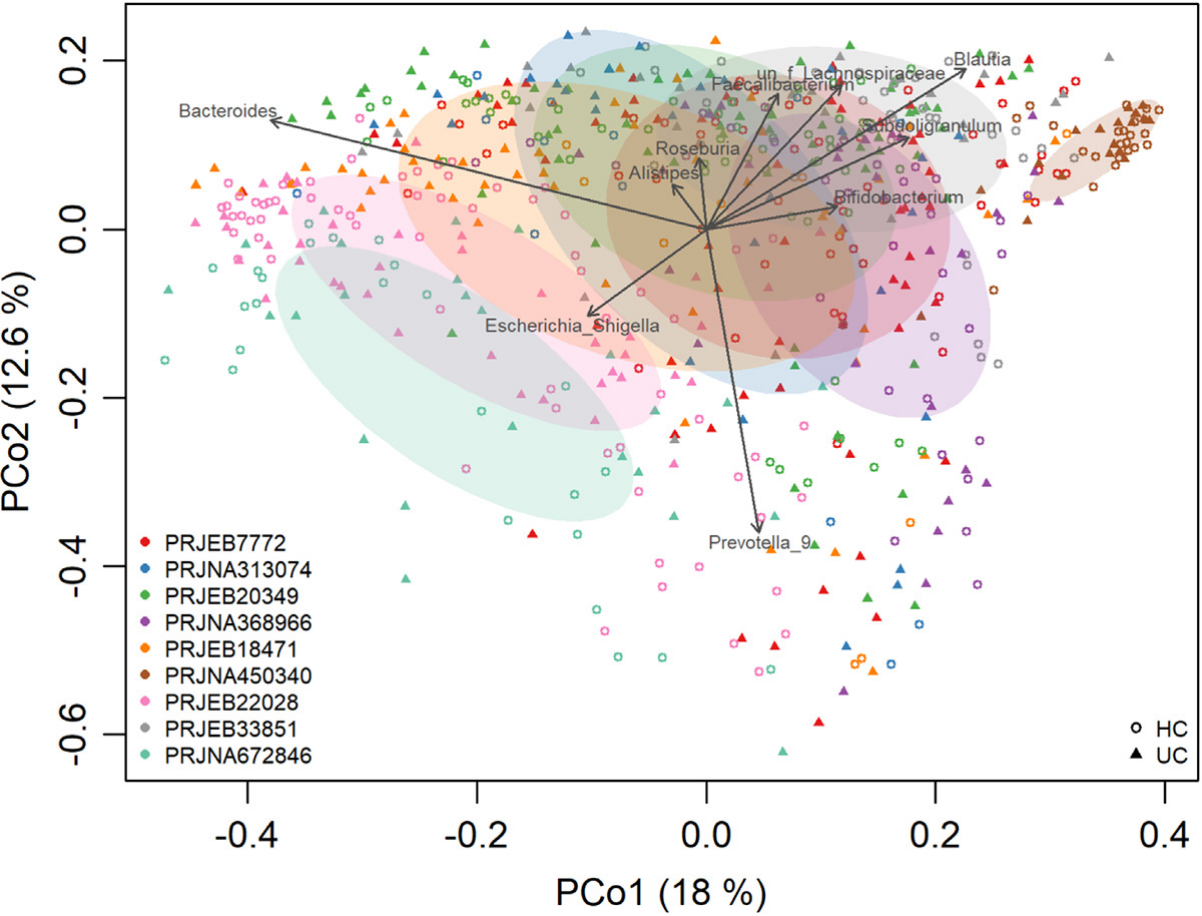
Microbial variations from different studies, probably due to the variables of DNA extraction methods, PCR amplification conditions, sequencing platforms, etc. The shapes represent UC patients or healthy controls. The colors represent the different studies.

A total of 656 samples from 9 studies were retained after quality filtering, including 306 healthy control and 350 UC patients (Table 1). As expected, gut bacterial communities from the 9 studies were all dominated by *Bacteroidetes* and *Firmicutes* (see Fig. S2 in the supplemental material). When all samples were combined, the overall gut microbial communities were significantly different between UC patients and healthy individuals (permutational multivariate analysis of variance [PERMANOVA], *P* < 0.001) and were significantly associated with the top abundant genera, including *Bacteroides*, and *Prevotella_9* (Fig. 2). However, it is also obvious that samples examined by principal-coordinate analysis (PCoA) were clustered primarily by individual studies (Fig. 2), showing the heterogeneity of microbiota across studies. This heterogeneity was probably due to strong variables of ethnicity and technical differences, including DNA extraction methods, PCR amplification conditions, and sequencing platforms adopted by individual studies (Table 1), and highlights the need for meta-analysis.

**TABLE 1:** Characteristics of the fecal 16S rRNA gene sequencing studies analyzed in this study

SRA no.	Reference or source	Country	No. of samples from:		DNA extraction method or kit	Region(s)	Sequencing platform
			HCs	UC patients			
PRJEB7772	Norman et al. (41)	USA	50	64	Qiagen		HiSeq
PRJNA313074	SRA_2016	USA	13	30			MiSeq
PRJEB20349	Paramsothy et al. (9)	Australia	55	64	MoBio PowerViral environmental RNA/DNA isolation kit	V1–V3	MiSeq
PRJNA368966	Bajer et al. (44)	Prague	31	29	MasterPure kit	V3–V4	MiSeq
PRJEB18471	Halfvarson et al. (46)	USA	9	53	MoBio Powersoil DNA kit	V4	HiSeq
PRJNA450340	Forbes et al. (45)	Canada	23	20	ZR-96 fecal DNA kit	V4	MiSeq
PRJEB22028	Zhou et al. (47)	China	66	40	Tiangen stool DNA kit	V4	MiSeq
PRJEB33851	SRA_2019	USA	34	25			NextSeq 500
PRJNA672846	SRA_2020	China	25	25	Qiagen fast stool minikit	V3–V4	MiniSeq

### Microbiome profile differences between UC and controls.

**FIG 3:**
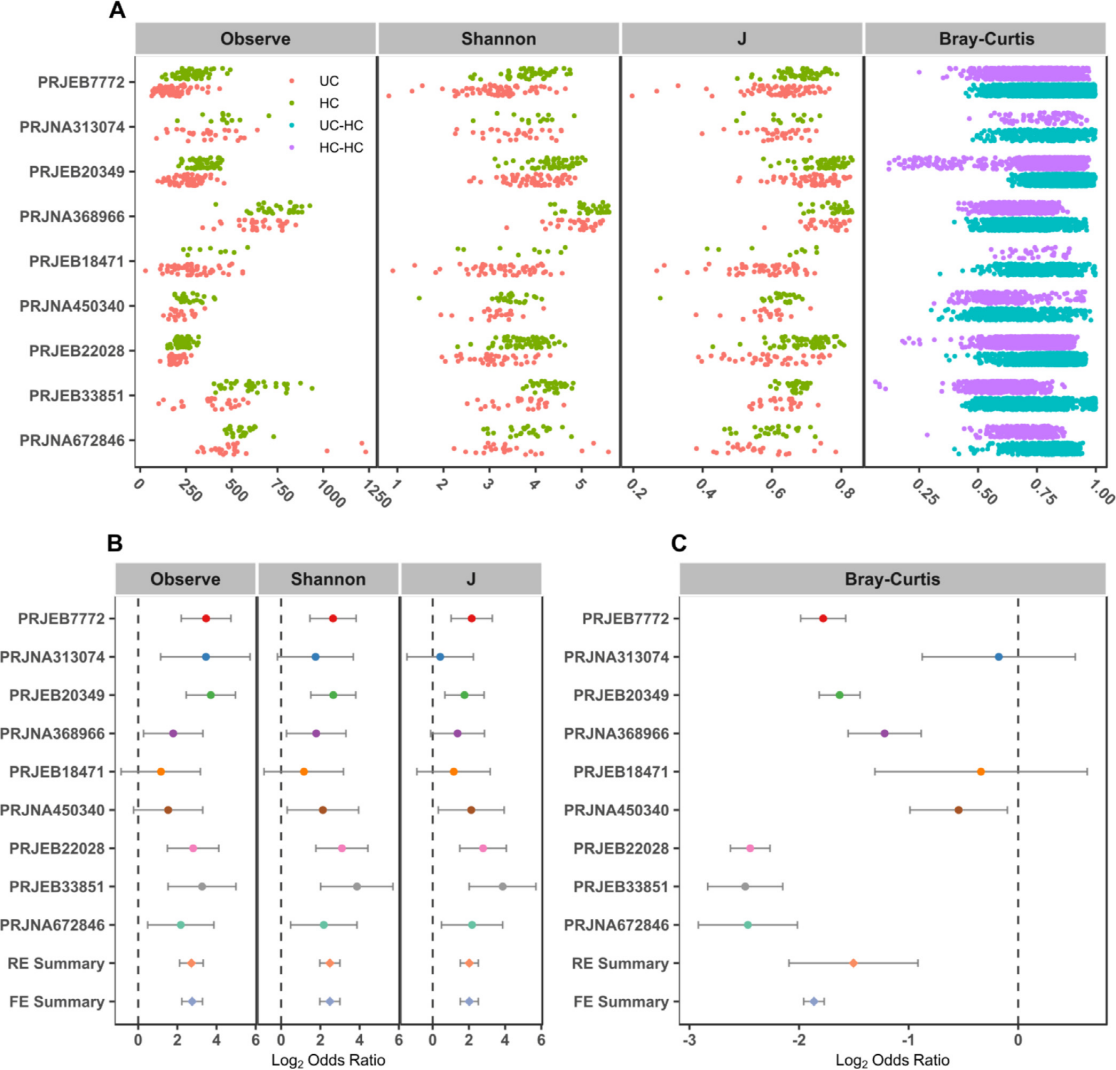
α-diversity metrics and Bray-Curtis distances between the individuals with UC and HCs. Comparison of observed OTUs (Observe), Shannon diversity, and evenness (J) between UC patients and HCs in 9 studies as well as distances of UC patients to HCs and HCs to HCs (A). (B and C) Random forest plots of the α-diversity metrics (B) and Bray-Curtis distances between individuals with UC and HCs (C). The error bar depicts the 95% confidence interval. A value of <1.0 (left side of the dashed line) indicates that the metric is higher in the UC patient than the control. A value of >1.0 (right side of the dashed lines) indicates that the metric is lower in the UC patient than the control. There was a significant difference between the case and the control if there was no crossing between the dashed line and the error bar.

Differences in α-diversity metrics (richness, Shannon diversity, and evenness) were first compared between UC and controls. We found significantly higher Shannon and evenness (J) indexes in healthy individuals than in those with UC in 7 of 9 studies and significantly higher microbial richness in controls in 8 of 9 studies (Fig. 3A and Table S3a). Due to the absence of consistent differences in individual studies, we calculated the odds ratios (ORs) of α-diversity. The ORs of all α-diversity metrics were significantly higher than 1.0 for UC in both the random effects (RE) model and fixed effects (FE) model with low heterogeneity (Fig. 3B), indicating significant lower microbial α-diversity in UC patients than controls. To measure β-diversity, we calculated a Bray-Curtis distance matrix for each data set and tested the significance by PERMANOVA. We found a significant difference in entire community between UC and controls in 8 of 9 studies (Table S3b). Again, by calculating the ORs based on the Bray-Curtis metric in each study, we found the significant bacterial community differences between UC and controls in both RE models and FE models with high heterogeneity (Fig. 3C). These results showed that there were dependable and significant community-wide changes in the bacterial community structures of UC patients.

**FIG 4:**
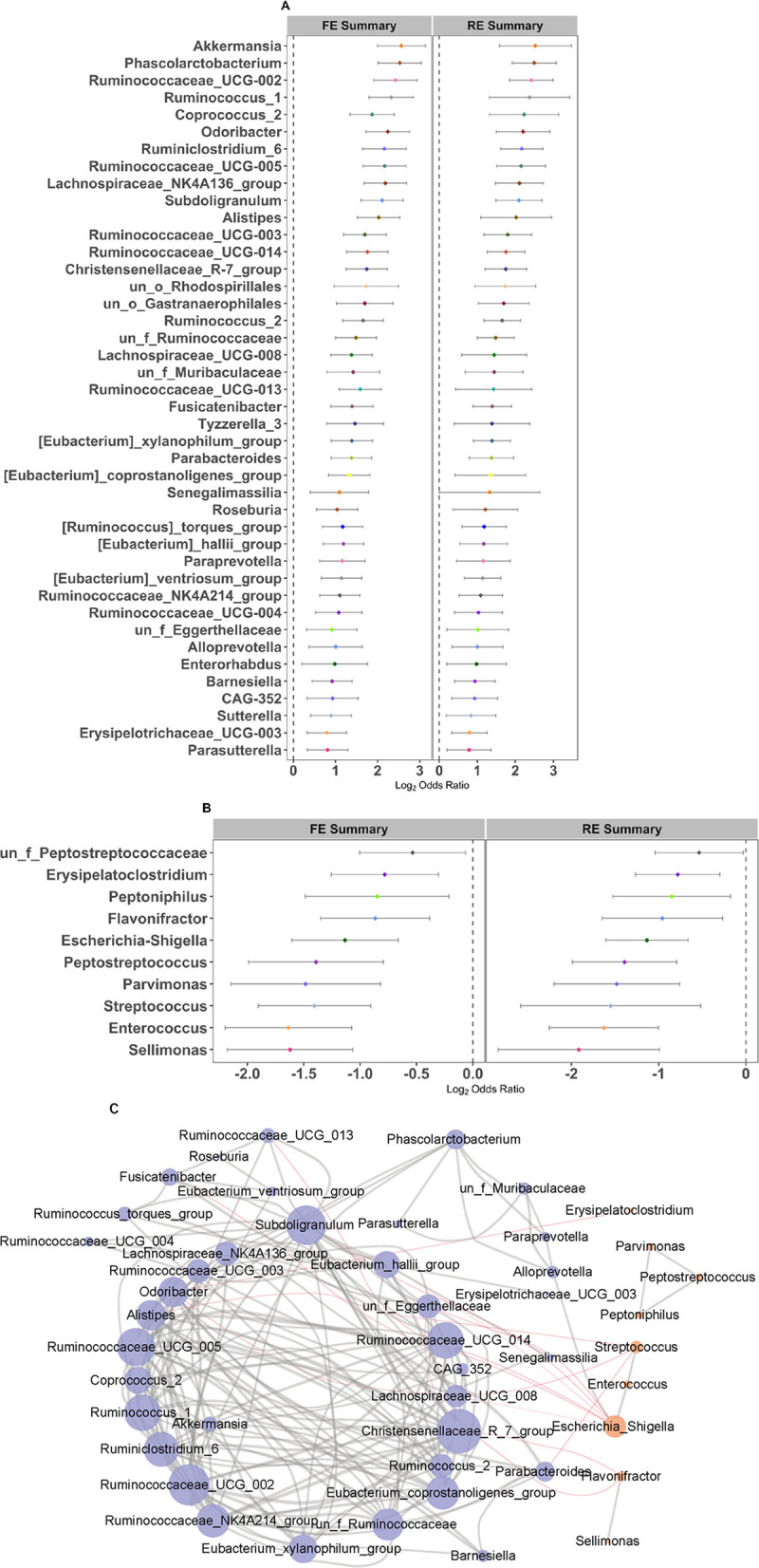
Random forest plots and network of genera with significant ORs. (A and B) Random forest plots of genera with significant ORs of >1 (A) and ORs of <1 (B) in RE and FE models. The error bar depicts the 95% confidence interval. A value of <1.0 (left side of the dashed line) indicates that the metric is higher in the UC patient than the control. A value of >1.0 (right side of the dashed lines) indicates that the metric is lower in the UC patient than the control. There was a significant difference between the case and the control if there was no crossing between the dashed line and the error bar. (C) Network of genera with significant ORs (Spearman's rank correlation, |*r*| > 0.3 and *P* < 0.05). Nodes with an OR of >1 and OR of <1 are in purple and orange, respectively. Positive and negative correlations (edges) are in gray and red, respectively.

In order to further identify the significantly different taxa and predicted pathways between UC patients and the healthy controls without UC, we calculated the ORs based on the common genera and pathways in each study. By quantifying the ORs, a total of 52 genera and 74 pathways had significant ORs for the UC patients (Fig. 4A and Table S4). Of these genera, most were depleted in UC patients (ORs bigger than 1.0 in both RE and FE models) compared to healthy controls (HCs), including the well-known protective taxa *Akkermansia*, *Alistipes*, and *Odoribacter*. Ten genera had significant ORs lower than 1.0 (enriched in UC), four of which were generally thought to be harmful for human health, including *Enterococcus*, Escherichia*-Shigella*, *Peptostreptococcus*, and Streptococcus (Fig. 4B). They were indeed negatively correlated with those genera with ORs larger than 1.0 in the co-occurrence network (Fig. 4C). Twenty-one pathways were depleted in UC patients, including acetyl coenzyme A (acetyl-CoA) fermentation to butanoate II (PWY-5676) to potentially produce anti-inflammatory short-chain fatty acids (SCFAs). Fifty-three pathways were enriched in UC patients, including enterobacterial common antigen biosynthesis (ECASYN-PWY), enterobactin biosynthesis (ENTBACSYN-PWY), peptidoglycan biosynthesis II (staphylococci) (PWY-5265), peptidoglycan biosynthesis V (and β-lactam resistance) (PWY-6470), and the superpathway of (Kdo)2-lipid A biosynthesis (KDO-NAGLIPASYN-PWY) (Table S4). As expected, significantly negative correlations were obtained for pathways enriched (OR < 1) and depleted (OR > 1) in UC (Fig. S3). Furthermore, a more complex network with higher-average neighbors was observed in predicted pathways (*n* = 20) than in genera (*n* = 8), implying the importance of microbial metabolism.

### Fecal bacterium-based classifier and important features.

**FIG 5:**
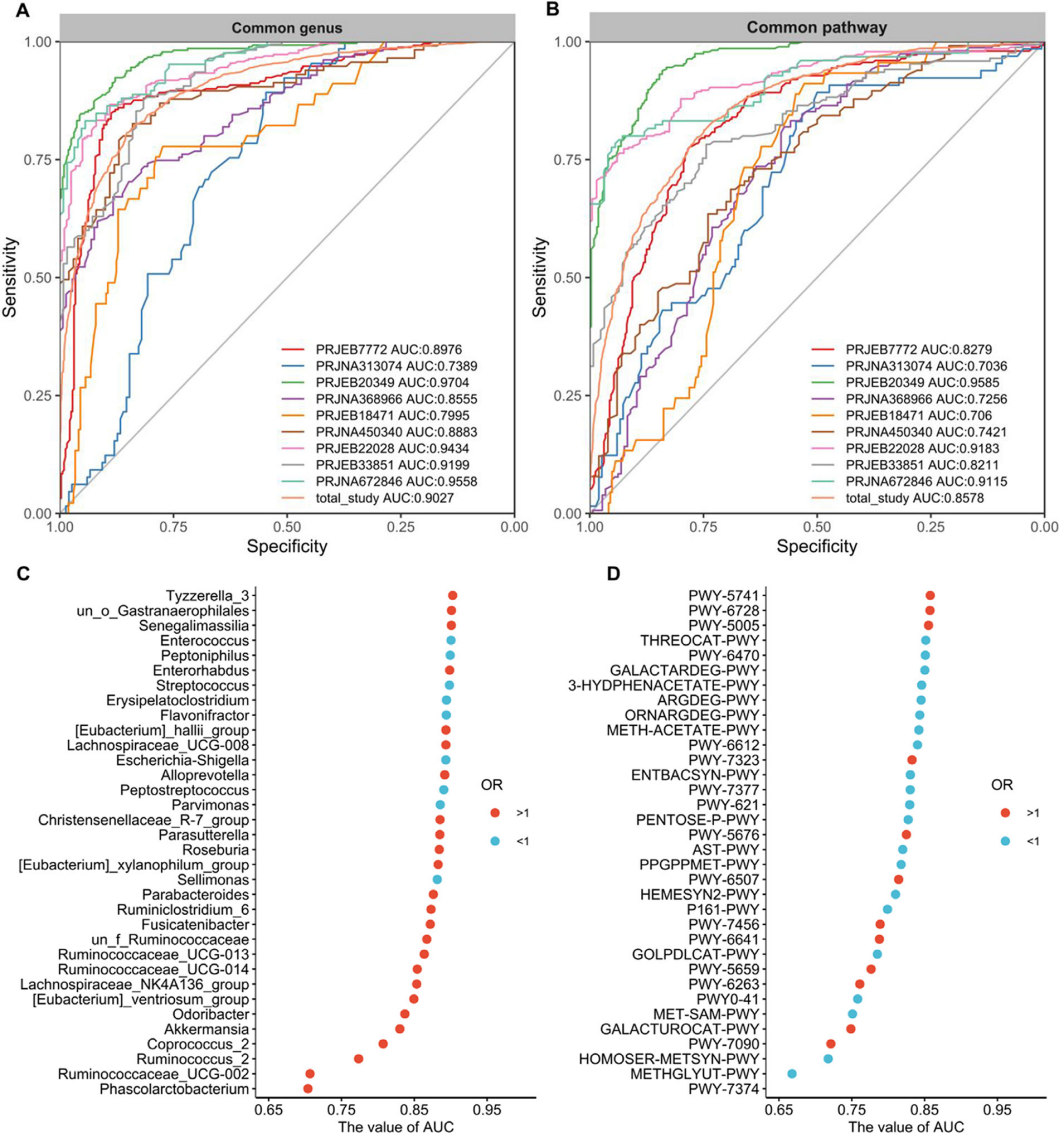
(A and B) ROC curves of individual studies and all studies based on the selected genera (A) and selected pathways (B). The gray lines represent the random predictors. The other lines depict the ROC curves of each study or all studies together using the cross-validation with five repeats. (C and D) Cumulative values of AUC for the selected 34 genera (C) and 34 pathways (D) by two-step feature selection. The red circle represents an OR of >1, and the blue circle represents an OR of <1.

Subsequently, we built classification models with the widely used and robust random forest (RF) algorithm based on genera or pathways with significant ORs for UC and to further refine important genera and pathways. As expected, we obtained RF models with comparable levels of performance, except for those in the PRJNA313074 and PRJEB18471 studies, likely due to the unbalanced samples. The median ± standard deviation (SD) sensitivity and specificity of the 9 individual studies for detection based on the cross-validation set using the common genus were 83.5% ± 7.4% and 82.9% ± 11.7% (area under the concentration-time curve [AUC] = 88.5% ± 7.6%), respectively, after feature selection. Meanwhile, we plotted the probability distribution based on the cross-validation results of the RF model for each study and found that values of UC patients and HCs were clearly separable and significantly (*P* < 0.001) different in both common genera (Fig. S4A) and pathways (Fig. S4B). More importantly, when pooling all studies, we obtained good performance based on genera (AUC = 0.90) and pathways (AUC = 0.86) for classification of UC (Fig. 5A and B) and further verified the models based on data from an additional study (PRJNA438164) with favorable generalization ability on both genera (AUC = 0.89) and pathways (AUC = 0.87) as well (Fig. S5A and B). The improvements in AUC values based on genera and pathways selected by feature selection are shown in Fig. 5C and D, respectively. Ultimately, the 34 genera and 34 pathways were obtained by two-step feature selection based on the pooled data set and were used for further analysis.

### Donor-recipient matching models and verification.

Six representative microbial characteristics (richness, distance, beneficial taxa, harmful taxa, beneficial pathways, and harmful pathways) from meta-analysis functioned as indicators to build a donor-recipient matching model by AHP. The pairwise comparison matrix of the criteria according to the experts' suggestion is shown in Table S5. The value of the consistency ratio was less than 0.1, which was within acceptable limits. Finally, the results of AHP showed that α-diversity was the most important indicator, with a weight of 28%. The overall α-diversity of donors was also higher than that of patients. Beneficial taxa and pathways had weights of 24% and 22%, respectively. The weights of indictors, including Bray-Curtis distances, harmful taxa, and harmful pathways were lower than 13% (Table S5).

**FIG 6:**
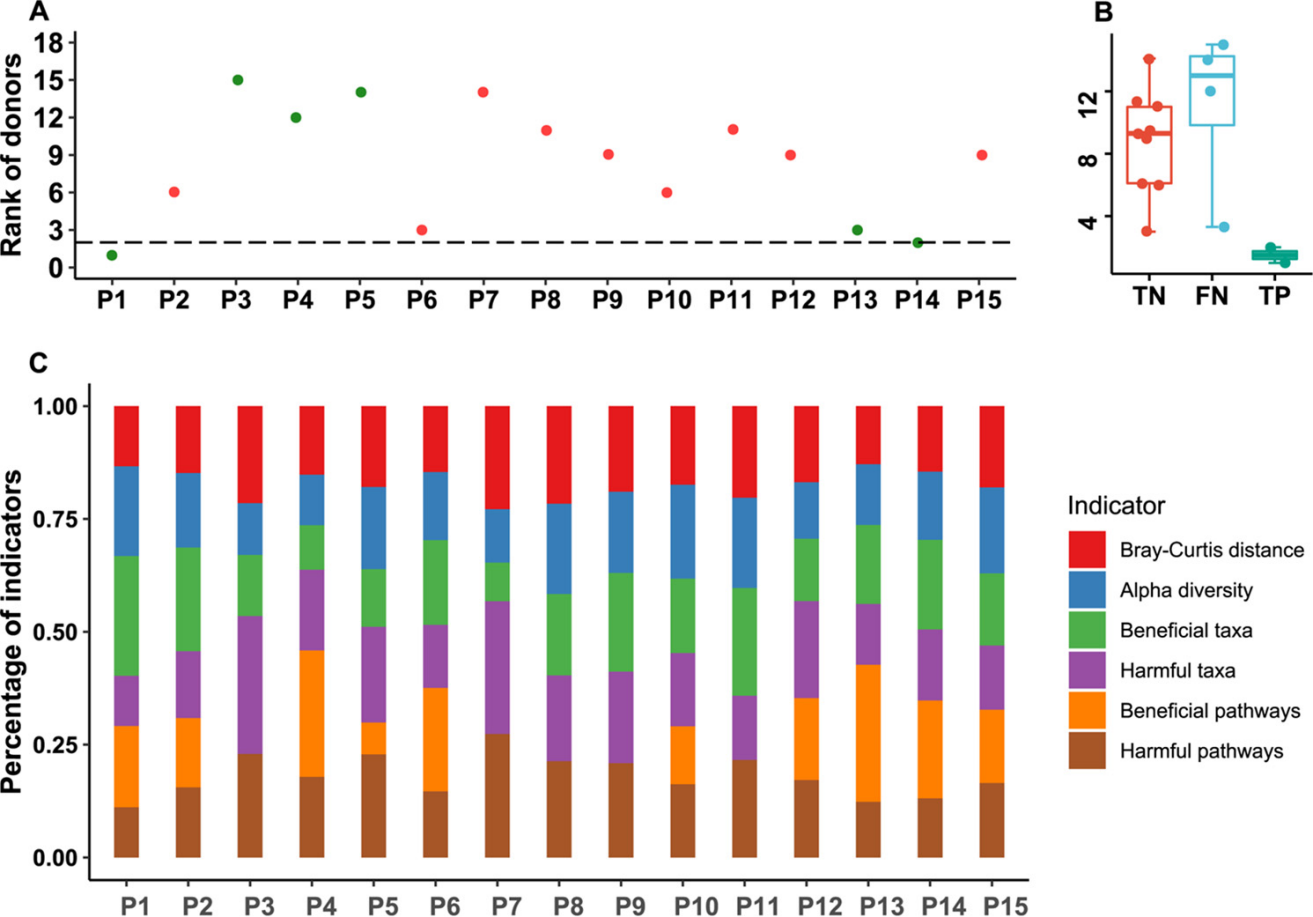
Verification of the donor-recipient matching model using a previous FMT clinical trial (PRJEB33851) for UC. (A) Model-recommending rank of the donors practically used for UC in the clinical trial. Green and red indicate that the patient achieved and did not achieve a clinical response after FMT, respectively. The dashed line represents the rank cutoff. The patient should get a response if the rank of the donor practically used in the FMT is below the cutoff. (B) Performance summary of the donor-recipient matching model. TP/TN represents the number of UC patients who got response/nonresponse in clinical trial and was predicted to get a response/nonresponse. FP/FN represents the number of UC patients who achieved nonresponse/response in clinical trial and was predicted to achieve a response/nonresponse. (C) Percentage of each indicator score relative to the total score for the donor practically used in the FMT of the clinical trial calculated from the model.

For verification of the model, we downloaded and analyzed sequencing data of two previous FMT clinical trials. Using the six gut microbial indicators of patients and donors as inputs, the model returned rank order for each patient (Fig. 6A and Fig. S6A). To optimize a threshold of donor rank for calling effectiveness, we tested the cutoff value from 1 to the number of all donors and evaluated the model performance by positive predictive value (PPV), specificity (Sp), accuracy (Acc), negative predictive value, and sensitivity. The results showed that the model had the best performance (highest PPV, Sp, and Acc) when the cutoff was set to 2 in both trials (Fig. S7), which was chosen as the final threshold. The accuracies of the donor-recipient matching models of the two studies were 73.33% and 71.43% compared to the clinical response of 15 patients from the PRJEB33851 study and 7 patients from the PRJNA438164 study (Fig. 6B and Fig. S6B). Also, we found that the patients received FMT from donors who were also recommended by the matching model were all effective in clinical studies PRJEB33851 (P1 and P14) and PRJNA438164 (P4 and P5).

## DISCUSSION

In this study, we conducted a comprehensive meta-analysis based on a wide collection of 16S rRNA gene sequencing data sets from 5 countries examined in a uniform manner to reveal the consistent differences in bacterial diversity, taxa, and predicted pathways between UC patients and healthy controls. By building the RF with step-by-step feature selection, we obtained models with good performances for classification of UC patients and healthy controls and further refined the important microbial features within the taxa and pathways. Finally, adopting the AHP method with bacterial diversity indexes, taxa, and pathways, we built a donor-recipient matching model to guide donor selection for a specified patient and verified its performance using a data set from an FMT clinical trial for ulcerative colitis. For the first time, this study presented a matching strategy integrating meta-analysis and an analytic hierarchy process to build a donor-recipient matching model to optimize donor selection for precision FMT in clinical studies and application.

While samples were integrated from 9 studies worldwide, significantly lower α-diversity indexes, including richness, Shannon diversity, and evenness, were observed in UC patients than in healthy controls by both RE and FE models. Lower microbial diversity is a common manifestation of gut dysbiosis associated with diseases (26) and was reported in previous studies involving UC patients (27, 28). Moreover, UC is characterized by the lack of beneficial taxa associated with SCFA production. Our results did show that UC patients harbor lower abundances (OR > 1) of *Alistipes*, *Odoribacter*, *Coprococcus*, etc., which are all associated with SCFA production (29). Another genus depleted in UC (OR > 1) is *Akkermansia*, which was shown to improve colitis with a reduction in infiltrating macrophages and CD8^+^ cytotoxic T lymphocytes in the colon via its outer membrane protein (30) and was shown to modulate immunological homeostasis by inducing IgG production and antigen-specific T cell response (31). Notably, a set of well-known harmful bacteria were found to be higher in UC samples (OR < 1), including Escherichia*-Shigella*, *Enterococcus*, and Streptococcus. Although an increasing number of studies are focusing the metabolism of UC (32–34), metabolic functions of microbiota associated with UC remained largely unknown. Based on the predicted pathways, we observed many pathways enriched or depleted in UC, such as higher contents of enterobacterial common antigen biosynthesis and enterobactin biosynthesis in UC. Enterobacterial common antigen is shared by all members of the *Enterobacteriaceae* and critical for the pathogenicity (35). Overall, these shifts and differences in the diversity, community composition, and metabolic pathways demonstrated significant dysbiosis of gut microbiota in UC patients compared to healthy controls.

Due to the dysbiosis of gut microbiota in UC patients, FMT from donors is adopted to modify and restore the gut microbial community perturbations. However, the donor and recipient gut microbiota compositions vary in ways that could impact the outputs of FMT trials (15, 36, 37), which could also explain the variations in response ratio of FMT in published UC studies (13, 14). This highlighted the demand for approaches to rationally select donors for patients before FMT to obtain better output, of which a donor-recipient matching model based on gut microbiota potentially represents a feasible protocol for precision FMT (17, 38). However, the biggest issues on a practical level are what should be considered in the matching process and how to set up such a matching model.

It has been reported that bacterial richness is an important indication for success in FMT (10, 19, 22, 39). Also, Kump et al. showed that higher richness of donor gut microbiota was associated with remission of UC (20), which is supported by our results that UC patients have lower richness indexes. In this situation, a donor who has more unique zero-radius operational taxonomic units (ZOTUs) than the patient does not have may provide more taxa to the patient and increase the richness, which is why richness was included and specifically designed as an indicator. In this study, significant differences detected in overall bacterial community between UC patients and HCs indicate that patient’s gut microbiota should be changed to a large extent to restore the balance. Therefore, a donor whose gut bacterial community is more dissimilar (greater bacterial distance) to the recipient has a better chance to successfully reshape the patient’s gut microbiota. Moreover, UC is thought as an absence/lack of beneficial taxa (OR > 1) and presence/overabundance of harmful taxa (OR < 1) (17), which is consistent with the results obtained in our meta-analysis. Additionally, some core functionality (e.g., SCFA production) was reported to be missing or enriched in UC or associated with the positive outcome of FMT (36), although only the abundance of SCFA producers in donor stool did not completely explain the responses (17). By the same logic as taxa, we treated all predicted pathways as beneficial ones when their ORs were >1 and as harmful pathways when their ORs were <1. Finally, six representative microbial features of richness, distance, beneficial taxa, harmful taxa, beneficial pathways, and harmful pathways were identified for the donor-receipt matching model to select donors with greater richness of unique taxa compared to patients, greater overall distance from patients, higher beneficial taxa and pathways, and lower harmful taxa and pathways. Through assigning weights by experts scoring and AHP method, donors with higher values from the model were ranked in the top hits for a specified patient. This model was successfully verified with favorable classification power for FMT effectiveness using data from the publicly available clinical studies.

Although a comprehensive analysis was conducted to build the donor-recipient matching model, there are significant limitations in this study. All data sets analyzed here are 16S rRNA gene-based bacterial profiles; therefore, this model did not consider the strain-level gut bacterial taxa nor other microbes—e.g., fungal microbiota (27) and virome (40, 41). The sequencing method adopted in these published studies measured the total bacterial communities and did not distinguish the viable bacteria from the injured or dead bacteria in the fecal samples (42). It is not clear how much fecal microbiota (quantity) is sufficient to effectively treat UC, even though the bacterial profile (composition) is considered in this model. This is an important parameter for precision FMT. Other aspects, including weights of each taxon/pathway in the beneficial or harmful taxa/pathway indicator (tuning by statistical significance or effect size), delivery model, transplantation frequency, and host genetic factors are also needed to be addressed for different diseases in the future. Even with these limitations, this study provides an example to leverage existing data sets to build donor-recipient matching models for precision FMT for UC as well as other diseases.

In conclusion, by meta-analysis of raw 16S rRNA gene sequencing data of 350 UC and 306 HC samples from 9 studies, we identified the unbalanced fecal bacterial indicators, including the significantly lower bacterial richness, evenness, and Shannon diversity in UC, significant differences in overall communities, and 34 genera and 34 predicted pathways with significant odds ratios and classification potentials for UC patients. By integrating these indicators and an analytic hierarchy process, we set up a bacterial community-based donor-recipient matching model for donor selection for patients with UC and further verified its performance (>70%) using microbial data sets from two FMT clinical trials for UC. More encouragingly, treatments of patients who received FMTs from donors who were recommended by the matching model were all effective in clinical practice. However, we must be aware of that this matching model as an exploratory hypothesis requires more samples, especially prospective trials for verification. Taken together, this study adopting the strategy of meta-analysis and an analytic hierarchy process developed and verified a donor-recipient matching model to optimize donor selection for patients with UC in FMT clinical studies and application. Moreover, we hypothesize that leveraging of public microbial data sets to build donor-recipient matching models for donor selection is necessary and feasible to ensure that FMT has higher effectiveness and successfully translates into clinical impact.

## MATERIALS AND METHODS

### Database search and study selection.

We performed a systematic literature search of the PubMed, Web of Science, and EMBASE databases up to May 2020 using the following terms: “ulcerative colitis (UC) and fecal microbiota and human and 16S rRNA” according to the “Preferred Reporting Items for Systematic Reviews and Meta-Analyses (PRISMA)” guidelines (43) (Fig. 1A). The abstracts identified in this search were screened to eliminate clearly irrelevant studies. After the review of the titles and abstracts, 17 publications were selected for a further review of the full texts (see Table S1 in the supplemental material). The criteria for study inclusion were as follows: (i) case (UC) − control (healthy) studies based on human fecal samples; (ii) samples sequenced by next-generation sequencing (NGS) for the 16S rRNA gene; and (iii) sequences, barcodes, and metadata publicly available or provided by authors until 20 May 2020 upon request by email. Studies involving case reports, reviews, and duplicate publications were excluded. At last, we obtained sequence data sets and metadata from 9 studies (Table 1) meeting the criteria (9, 41, 44–47): 7 studies associated with gut microbiota of UC were excluded due to incomplete information on sequences, barcodes, or metadata (20, 48–51).

### Microbiome data processing.

The sequencing data included in our analysis were mainly generated by Illumina MiSeq. The most frequently sequenced hypervariable region was V4 or the V3-V4 regions of the 16S rRNA gene (Table 1). Since different cohorts adopted different hypervariable regions and different sequencing platforms, it was essential to analyze the data sets in a uniform manner to minimize the impact of these differences in the following analyses. For paired-end reads, they were assembled using FLASH by default parameters, except with -x 0.2 and -M 200 for the V3-V4 regions and -M 150 for the V1-to-V3 and V4 regions. After that, on this basis, we uniformly used Usearch with the parameter -fastq_maxee 0.5 for quality filtering again. Reads were denoised into zero-radius operational taxonomic units (ZOTUs), and chimeras were filtered with UNOISE3 (52). Samples that failed to yield at least 10,000 sequences were removed, except for two studies (9, 47) in which samples with less than 3,000 sequences were removed due to the poor sequencing quality. All analyses were performed on ZOTU tables rarefied to the lowest sequencing depth within each study. Taxonomic assignment of ZOTUs was performed in QIIME 1.9.1 (http://qiime.org/) (53) using the QIIME feature classifier plugin and the SILVA132 sequence database. Microbial function was predicted by PICRUSt2 (54).

### Statistical analysis.

Microbial α-diversity was measured using richness (observed OTUs [Obs]), Shannon diversity, and evenness J based on rarefied ZOTU tables (55). Significant differences in α-diversity indexes between UC patients and healthy controls were determined using the Wilcoxon test. Differences in overall communities (β-diversity) were calculated using the Bray-Curtis distance metric and visualized by principal-coordinate analysis (PCoA) plots using custom R scripts. Significant differences in β-diversity across patient groups were evaluated using permutational multivariate analysis of variance (PERMANOVA) with 10^4^ permutations in the vegan package (56). envfit() from the vegan package was used to fit the top 10 genera in relative abundance to the PCoA unconstrained axis. All *P* values were then adjusted using the false-discovery rate (FDR) correction. All statistical analyses were conducted in R v.3.5.3 (57). The network was generated by Cytoscape v.3.5.1. Other figures were plotted using the ggplot2 v.3.0.0 (58) and gridExtra packages (59). Meta-analyses of bacterial α-diversity, β-diversity, taxa, and pathways of the 9 studies (Table 1) were performed to explore the consistent microbial features. Generally, we calculated the odd ratios (ORs) of these metrics by assigning any value above the overall median of the metric within the study as positive (60). The random effects model and fixed effects model were conducted in the metafor package (61) based on the contingency table generated from the median value.

Random forest (R package, caret) models were trained for individual studies and the data set combining all studies at the common genus or pathway levels to test whether a mixture of featured taxa can predict UC. We evaluated model performance using 5-fold cross-validation and scored the predictive power in a receiver operating characteristic (ROC) analysis. To further select important bacteria taxa and pathways, we trained random forest models based on genera and pathways by two-step feature selection. First, we calculated the AUC values of individual genera and pathways. Then, on the basis of the rank of the AUC values, we executed step-by-step feature selection to generate final model (detailed in the supplemental material). The genera and pathways that overlapped in meta-analysis and ROC analyses were recognized as the more important features for donor selection in downstream analyses.

### Donor-recipient matching model by AHP.

Based on results obtained from the meta-analysis on bacterial communities in this study, a total of 6 metrics with significant differences between UC patients and HCs or with significant importance for differentiating UC patients and HCs in RF models were recognized as the criteria of the donor-recipient matching model for donor selection. These 6 metrics were assigned to diversity indicators (α-diversity and distance), individual taxon indicators (beneficial taxa and harmful taxa), and predicted pathway indicators (beneficial pathways and harmful pathways). The following are the detailed statements of these indicators.

Diversity indicators included subindicators of α-diversity that considered the donor-specific ZOTUs (equation 1), the cumulative relative abundance of these ZOTUs (equation 2), and the overall Bray-Curtis distance between the donor and patient (equation 3).

**(i) α-diversity indicators.** The α-diversity indicators were calculated as shown in equations 1 and 2:

(1) $${\text{Obs}}_{\alpha -\text{diver}}={\text{Obs}}_{\text{Donor}}-{\text{Obs}}_{{\text{Donor}}^{\cap \;\;}\text{patient}}$$

(2) $${\text{Abu}}_{\alpha -\text{diver}}={\text{Abu}}_{{\text{Obs}}_{\alpha -\text{diver}}}$$

Obs_α-diver_ represents the richness of donor-specific ZOTUs, Obs_Donor_ indicates the bacterial richness of the donor, Obs_Donor∩patient_ represents the number of ZOTUs shared between donor and patient, and Abu_Obs_α-diver__ is the cumulative relative abundance of the donor-specific ZOTUs.

**(ii) Distance indicator.** The distance indicator was calculated as shown by equation 3:

(3) $$D_{\text{Bray}}=1\;-\;2\frac{\sum^{\;}\text{min}(S_{P,i},S_{D,i})}{\sum^{\;}S_{P,i}\;+\;\sum^{\;}S_{D,i}}$$

S_P,i_ and S_D,i_ represent the count of the *i*th OTU in the patient and donor communities, respectively.

Individual taxon indicators included subindicators of beneficial taxa (defined as ORs of >1) and harmful taxa (defined as ORs of <1). The absence of a beneficial taxon indicator constitutes Ratio.bt_Donor_, the ratio of the number of beneficial genera that the patient lacks and are detected in the donor to the number of beneficial genera that the patient lacks (equation 4), and the cumulative relative abundance of these genera (equation 5). Similarly, the presence of a harmful taxon indicator constitutes Ratio.ht_Donor_, the ratio of the number of harmful genera that are detected both in the patient and donor to the number of harmful genera that are detected in the patient (equation 6), and the cumulative relative abundance of these genera (equation 7).

**(iii) Absence of beneficial taxa.** The absence of beneficial taxa was calculated as shown by equations 4 and 5:

(4) $$\text{Ratio}{\text{.bt}}_{\text{Donor}}=\text{Genera}{\text{.b}}_{{\text{patient\_absence}}_{\text{Donor}}}\text{/Genera}{\text{.b}}_{\text{patient\_absence}}$$

(5) $$\text{Abu}{\text{.bt}}_{\text{Donor}}={\text{Abu}}_{\text{Genera}{\text{.b}}_{{\text{patient\_absence}}_{\text{Donor}}}}$$

Ratio.bt_Donor_ is the ratio of the number of beneficial genera that the patient lacks and are detected in the donor (Genera.b_patient_absence_Donor__) to the number of beneficial genera that the patient lacks (Genera.b_patient_absence_), and Abu.bt_Donor_ represents the cumulative relative abundance of Genera.b_patient_absence_Donor__.

**(iv) Presence of harmful taxa.** The presence of harmful taxa was calculated by equations 6 and 7:

(6) $$\text{Ratio}{\text{.ht}}_{\text{Donor}}=\text{Genera}{\text{.h}}_{{\text{patient\_presence}}_{\text{Donor}}}\text{/Genera}{\text{.h}}_{\text{patient\_presence}}$$

(7) $$\text{Abu}{\text{.ht}}_{\text{Donor}}={\text{Abu}}_{\text{Genera}{\text{.h}}_{{\text{patient\_presence}}_{\text{Donor}}}}$$

Ratio.ht_Donor_ is the ratio of the number of harmful genera that are detected in both the patient and donor (Genera.h_patient_presence_Donor__) to the number of genera that are detected in the patient (Genera.h_patient_presence_), and Abu.ht_Donor_ represents the cumulative relative abundance of Genera.h_patient_presence_Donor__.

The individual pathway indicator included subindicators of beneficial pathways (defined as ORs of >1) and harmful pathways (defined as ORs of <1). The absence of a beneficial pathway indicator consists of Ratio.bp_Donor_, the ratio of the number of beneficial pathways that the patient lacks and are detected in the donor to the number of beneficial pathways that the patient lacks (equation 8) and the cumulative relative abundance of these pathways (equation 9). Similarly, the presence of a harmful pathway indicator consists of Ratio.hp_Donor_, the ratio of the number of harmful pathways that are detected both in the patient and donor to the number of harmful pathways that are detected in the patient (equation 10) and the cumulative relative abundance of these pathways (equation 11).

**(v) Absence of beneficial pathways.** The absence of beneficial pathways was calculated as shown by equations 8 and 9:

(8) $$\text{Ratio}{\text{.bp}}_{\text{Donor}}=\text{Pathway}{\text{.b}}_{{\text{patient\_absence}}_{\text{Donor}}}\text{/Pathway}{\text{.b}}_{\text{patient\_absence}}$$

(9) $$\text{Abu}{\text{.bp}}_{\text{Donor}}={\text{Abu}}_{\text{Pathway}{\text{.b}}_{{\text{patient\_absence}}_{\text{Donor}}}}$$

Ratio.bp_Donor_ is the ratio of the number of pathways that the patient lacks and are detected in the donor (Pathway.b_patient_absence_Donor__) to the number of pathways that the patient lacks (Pathway.b_patient_absence_), and Abu.bp_Donor_ represents the cumulative relative abundance of Pathway.b_patient_absence_Donor__.

**(vi) Presence of harmful pathways.** The presence of harmful pathways was calculated as shown by equations 10 and 11:

(10) $$\text{Ratio}{\text{.hp}}_{\text{Donor}}=\text{Pathway}{\text{.h}}_{{\text{patient\_presence}}_{\text{Donor}}}\text{/Pathway}{\text{.h}}_{\text{patient\_presence}}$$

(11) $$\text{Abu}{\text{.hp}}_{\text{Donor}}={\text{Abu}}_{\text{Pathway}{\text{.h}}_{{\text{patient\_presence}}_{\text{Donor}}}}$$

Ratio.hp_Donor_ is the ratio of the number of pathways that the patient lacks and are detected in the donor (Pathway.h_patient_presence_Donor__) to the number of pathways that the patient lacks (Pathway.h_patient_presence_), and Abu.hp_Donor_ represents the cumulative relative abundance of Pathway.h_patient_presence_Donor__.

**(vii) Calculation of the score of the donors.** The scores of the donors were calculated as shown by equation 12:

(12) $$\begin{array}{ll} {\text{Score}}_{\text{donor}}= & {\text{Weight}}_{\alpha -\text{diver}}\times {\text{Weight(Matrix}}_{\alpha -\text{diver}}\text{)}+{\text{Weight}}_{\text{Distance}}\\ & \times \text{Weight}\left({\text{Matrix}}_{\text{Distance}}\right)+{\text{Weight}}_{\text{Absence\_taxa}}\times {\text{Weight(Matrix}}_{{\text{Absence}}_{\text{taxa}}}\text{)}\\ & \;+{\text{Weight}}_{\text{presence\_taxa}}\times {\text{Weight}(\text{Matrix}}_{\text{presence\_taxa}})+{\text{Weight}}_{\text{Absence\_path}}\\ & \times {\text{Weight}(\text{Matrix}}_{\text{Absence\_path}})+{\text{Weight}}_{\text{presence\_path}}\times {\text{Weight(Matrix}}_{{\text{presence}}_{\text{path}}}\text{)} \end{array}$$

Weight_*i*_ *i* ∈ (α-diver, Distance, Absence_taxa_, presence_taxa_, Absence_path_, presence_path_) represents the weight of the six indicators calculated according to the judgment matrix, and Weight (*i*) *i* ∈ (Matrix_α-diver_, Matrix_Distance_, Matrix_Absence_taxa__, Matrix_presence_taxa_, Matrix_Absence_path_, Matrix_presence_path__) represents the weight ranking of each donor calculated according to the judgment matrix for each indicator. A pseudocode is provided in Fig. S1.

### Verification of the donor-recipient matching model.

Sequencing data from two previous clinical studies, PRJNA438164 (62) and PRJEB33851 (63), on treatment of UC with FMT were downloaded from NCBI database and analyzed to verify the applicability and accuracy of our donor-recipient matching model. In the clinical test, each UC patient (22 patients in both studies) received FMT from a specified donor (22 donors in total), and the patients were followed up and reviewed after FMT (Table S2). In our test, sequencing data from each patient and all donors within each study were used as input, and the output is a rank of donors that the model recommended. For evaluation of the model performance, we defined that a patient should reach a response if the donor factually used in the FMT was in the top 2 of the donor rank from the model (TP). On the contrary, a patient would be thought not to reach a response if the donor factually used in the FMT was not in the top 2 of the donor rank (TN). The ratio of the sum of TP and TN to the number of all patients within each study was calculated to show the effectiveness of the donor-recipient matching model.
