# Supplementary material for: A Matching Strategy To Guide Donor Selection for Ulcerative Colitis in Fecal Microbiota Transplantation: Meta-Analysis and Analytic Hierarchy Process
Source: Microbiol Spectr. 2022 Dec 6;11(1):e02159-21. doi: 10.1128/spectrum.02159-21 (PMC9927247; doi:10.1128/spectrum.02159-21)
Supplement: Supplemental file 1 — Fig. S1 to S7. Download spectrum.02159-21-s0001.pdf, PDF file, 1.0 MB [file spectrum.02159-21-s0001.pdf]

Supporting Information for:

A matching-strategy to guide donor selection for ulcerative colitis in fecal microbiota transplantation: meta-analysis and analytic hierarchy process

Supporting information:

In this study, we firstly determined these two main parameters ntree and mtry. The parameter ntree is the number of decision trees contained in the random forest, default is 500. The parameter mtry is the number of variables contained in each decision tree. The optimal mtry value was selected according to the OOB estimate value of error rate. The default parameters were used for the rest of the parameters. Secondly, for each research data set, a two-step feature selection method was adopted to screen features and remove redundant information. i) AUC values of the common genera or pathways in each study were calculated one by one based on RF through 5-fold cross-validation, and then were sorted in descending order. ii) Stepwise selection of candidate features based on AUCs of common genera or pathways. The feature was retained if the AUC value of the model increased after addition of the candidate genus or pathway. Otherwise, this candidate feature was removed. These remaining features were used to build the final model based on genera or pathways.

Supporting Figures:

Input:

Genera of the donor and recipient: $Genera_{donor}$, $Genera_{recipient}$

Observe OTUs of the donor and recipient: Obs_{donor}, $Obs_{recipient}$

Patnways of the donor and recipient: $Pathway_{donor}$, $Pathway_{recipient}$

Output:

The score of all donors: $\text{Score}_{\text{donor}}$

Step 1: Diversity indicators

Distance indicator: $D_{bray} = 1 - 2\frac{\sum \min(S_{P,i}, S_{D,i})}{\sum S_{P,i} + \sum S_{D,i}}$

Alpha-diversity indicator: $Obs_{alpha-diver} = Obs_{Donor} - Obs_{Donor \cap patient}$

$Abu_{alpha-diver} = Abu_{Obs_{alpha-diver}}$

Step 2: Individual taxa indicators

Absence of beneficial taxa:

$Ratio.bt_{Donor} = Genera.b_{patient\_absence_{Donor}} / Genera.b_{patient\_absence}$

$Abu.bt_{Donor} = Abu_{Genera.b_{patient_{absence_{Donor}}}}$

Presence of harmful taxa:

$Ratio.ht_{Donor} = Genera.h_{patient\_presence_{Donor}} / Genera.h_{patient\_presence}$

$Abu.ht_{Donor} = Abu_{Genera.h_{patient\_presence_{Donor}}}$

Step 3: Individual pathway indicators

Absence of beneficial pathways:

$Ratio.bp_{Donor} = Pathway.b_{patient\_absence_{Donor}} / Pathway.b_{patient\_absence}$

$Abu.bp_{Donor} = Abu_{Pathway.b_{patient_{absence_{Donor}}}}$

Presence of harmful pathways:

$Ratio.hp_{Donor} = Pathway.h_{patient\_presence_{Donor}} / Pathway.h_{patient\_presence}$

$Abu.hp_{Donor} = Abu_{Pathway.h_{patient\_presence_{Donor}}}$

Step 4: Calculate the weight of the indicators

$Weight(Matrix_{Distance}) = Weight(JudgeMatrix(D_{bray}))$

$Weight(Matrix_{alpha-diver}) = Weight(JudgeMatrix(Obs_{alpha-diver}))$

$Weight(Matrix_{Absence\_taxa}) = Weight(JudgeMatrix(Ratio.bt_{Donor}))$

$Weight(Matrix_{presence\_taxa}) = Weight(JudgeMatrix(Ratio.ht_{Donor}))$

$Weight(Matrix_{Absence\_path}) = Weight(JudgeMatrix(Ratio.bp_{Donor}))$

$Weight(Matrix_{presence_{path}}) = Weight(JudgeMatrix(Ratio.hp_{Donor}))$

Step 5: Calculate the score of the donors

$$Score_{donor} = Weight_{Distance} * Weight(Matrix_{Distance}) + Weight_{alpha-diver} * Weight(Matrix_{alpha-diver}) + Weight_{Absence\_taxa} * Weight(Matrix_{Absence_{taxa}}) + Weight_{presence\_taxa} * Weight(Matrix_{presence\_taxa}) + Weight_{Absence\_path} * Weight(Matrix_{Absence\_path}) + Weight_{presence\_path} * Weight(Matrix_{presence_{path}})$$

Fig S1. The pseudocode of Meta-AHP algorithm for the donor-recipient-matching model.

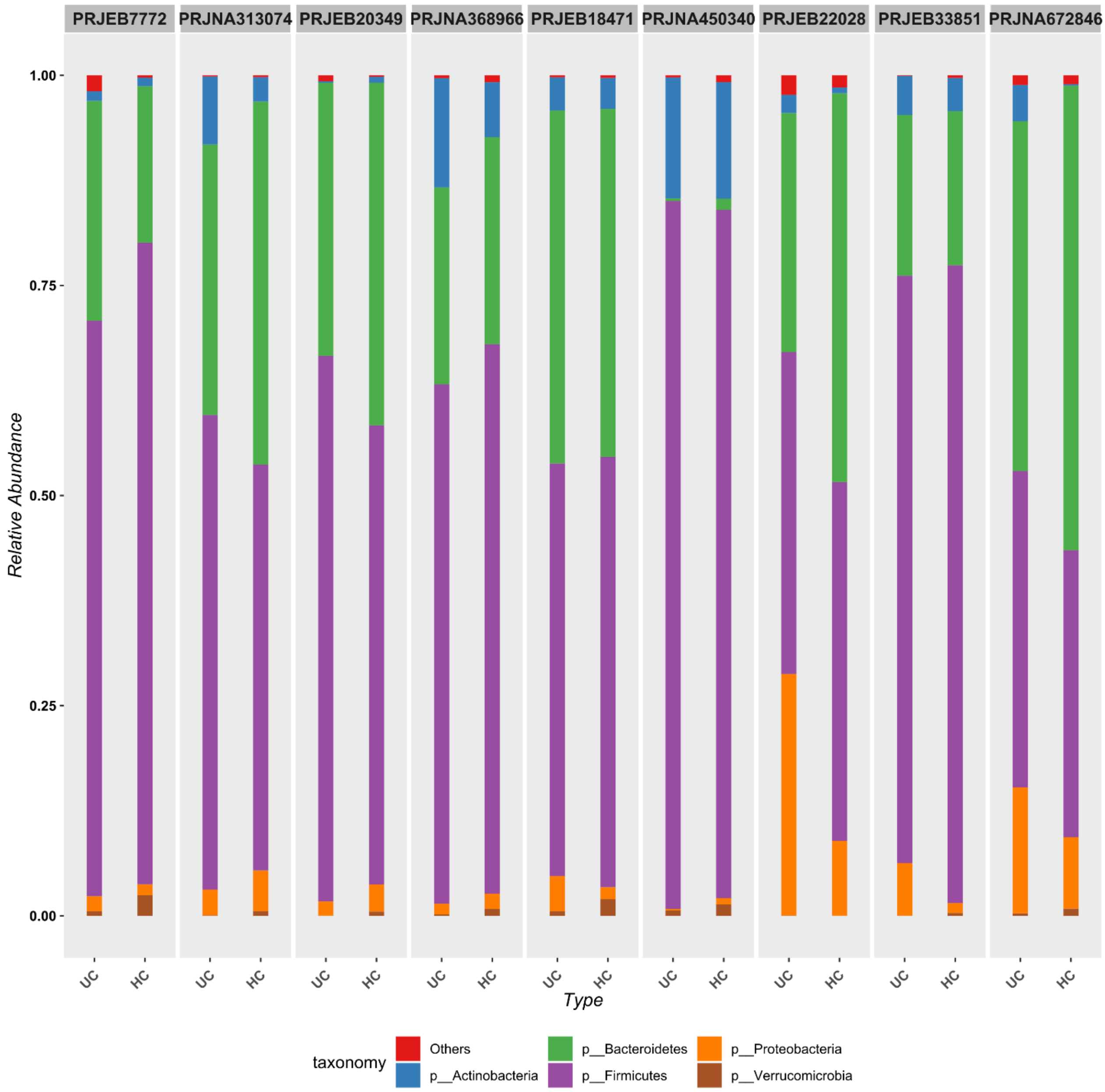

Fig S2. The relative abundance of bacteria phylum between UC and controls in different research projects.

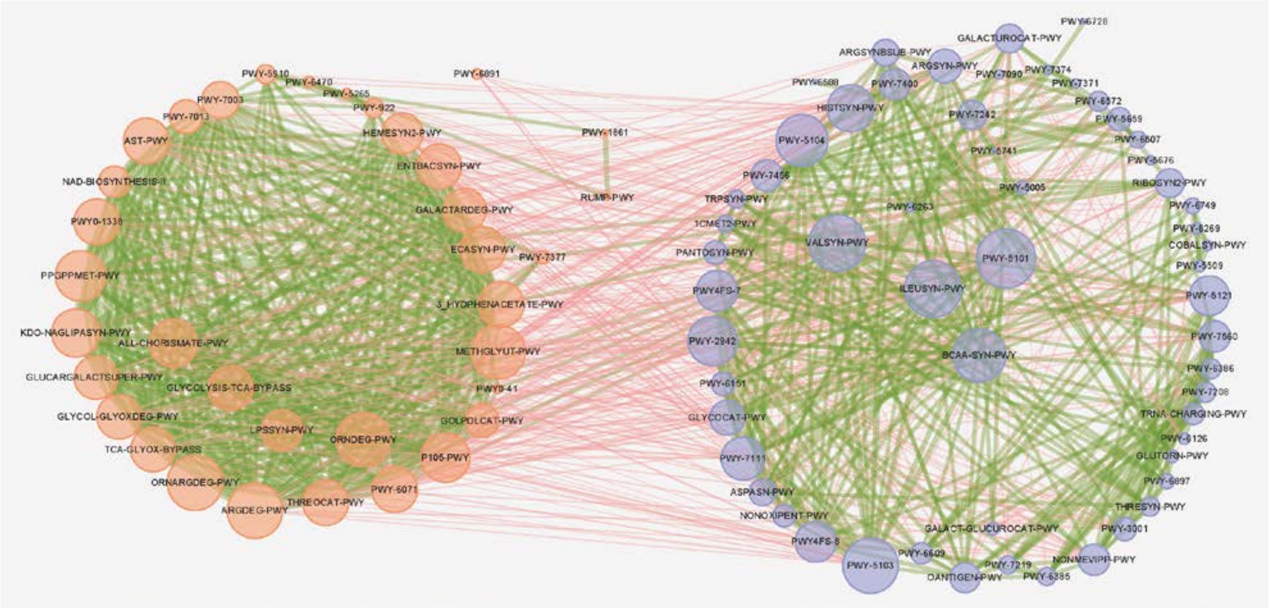

Fig S3. Network of pathways with significant ORs. Nodes with OR >1 and OR <1 were in purple and orange colors, respectively. Positive and negative correlations (edges) were in green and red colors, respectively.

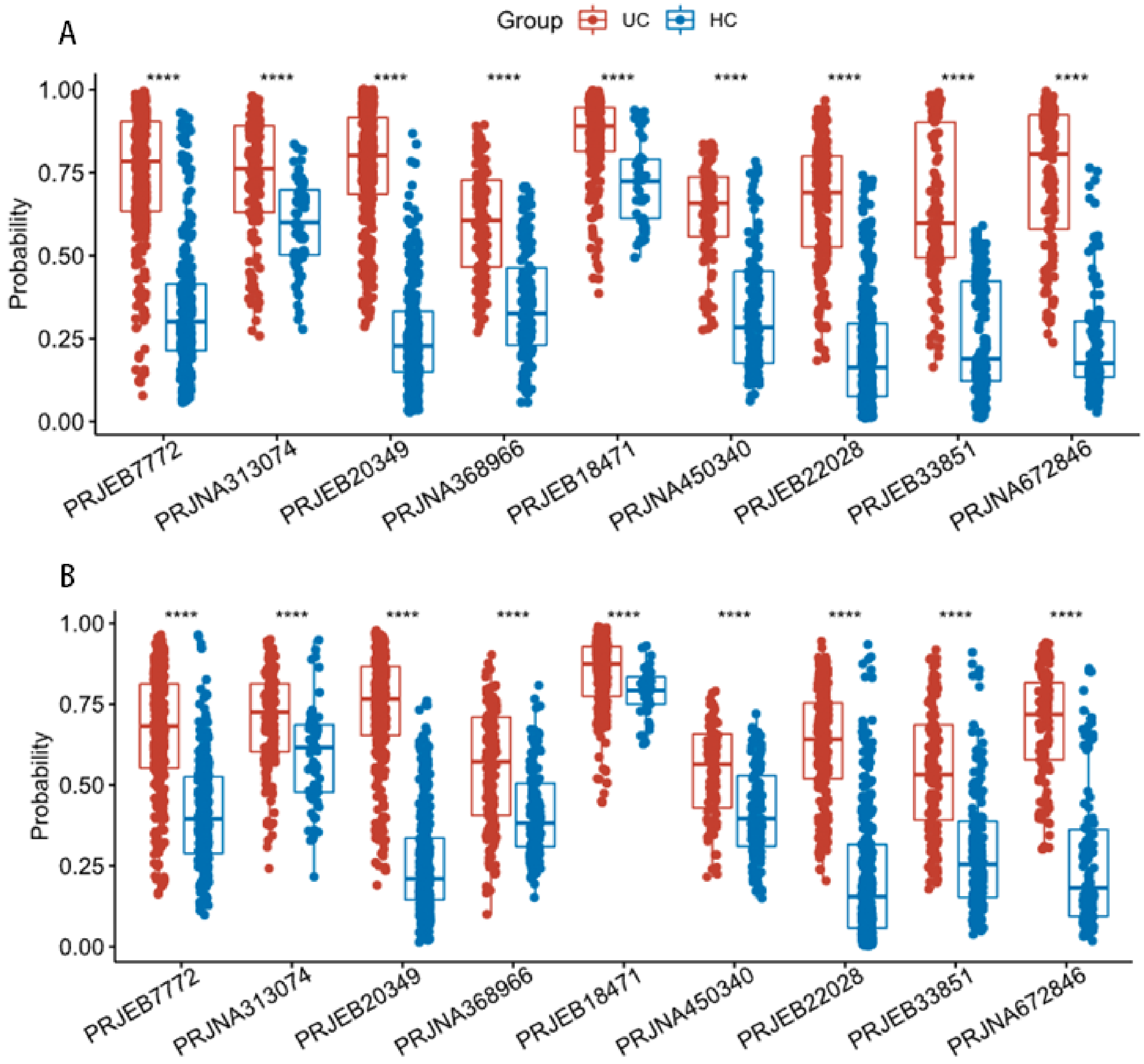

Fig S4. The probability distribution of predictions based on the cross-validation results of the RF model for each study in common genera (A) and common pathways (B).

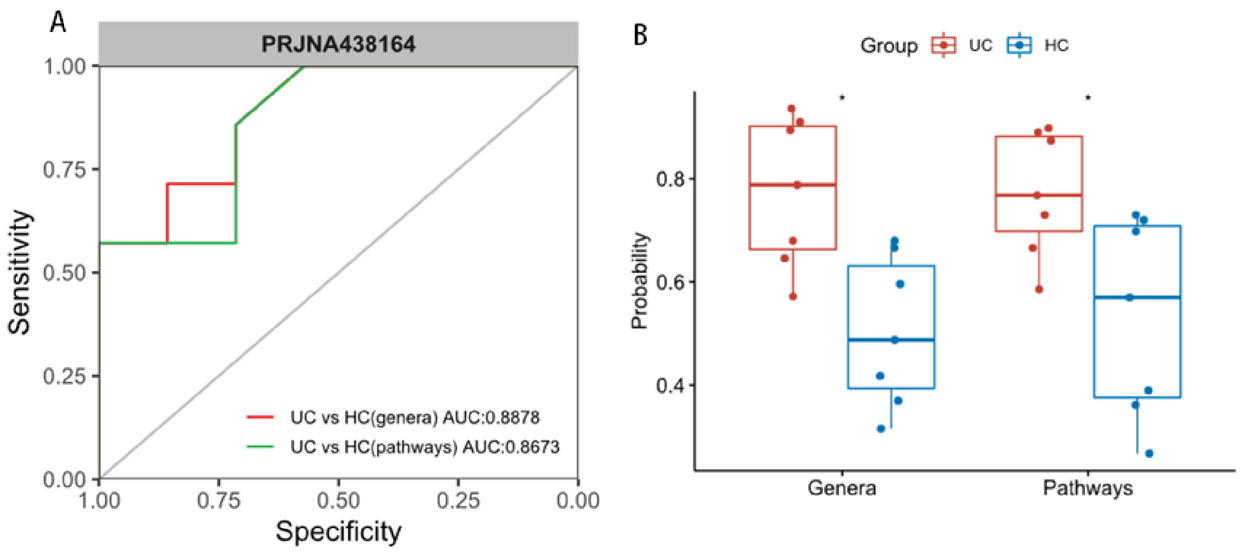

Fig S5. Verification of the pooled dataset models using data from a previous FMT clinical trial (PRJNA438164) for UC. (A)The ROC curves of models based on common genera and pathways. (B) The probability distribution of predictions based on the validation results for UC and HC.

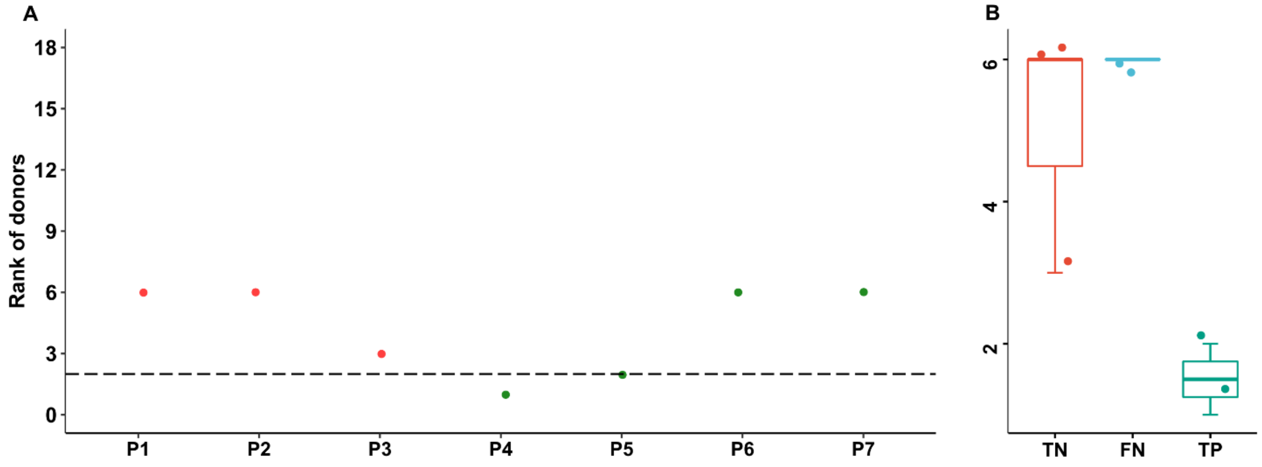

Fig S6. Verification of the donor-recipient-matching model using a previously FMT clinical trial (PRJNA438164) for UC. (A)The model-recommending rank of the donors practically used for UC in the clinic trial. The green and red colors represent the patient achieved and did not achieved clinical response after FMT, respectively. The dashed line represents the rank cutoff. The patient should get response if the rank of the donor practically used in the FMT is below the cutoff. (B) The performance summary of the donor-recipient-matching model. TP/TN represents the number of UC patients who got response/ non-response in clinical trial and was predicted to get response/ non-response. FP/FN represents the number of UC patients who achieved non-response/ response in clinical trial and was predicted to achieve response/ non-response.

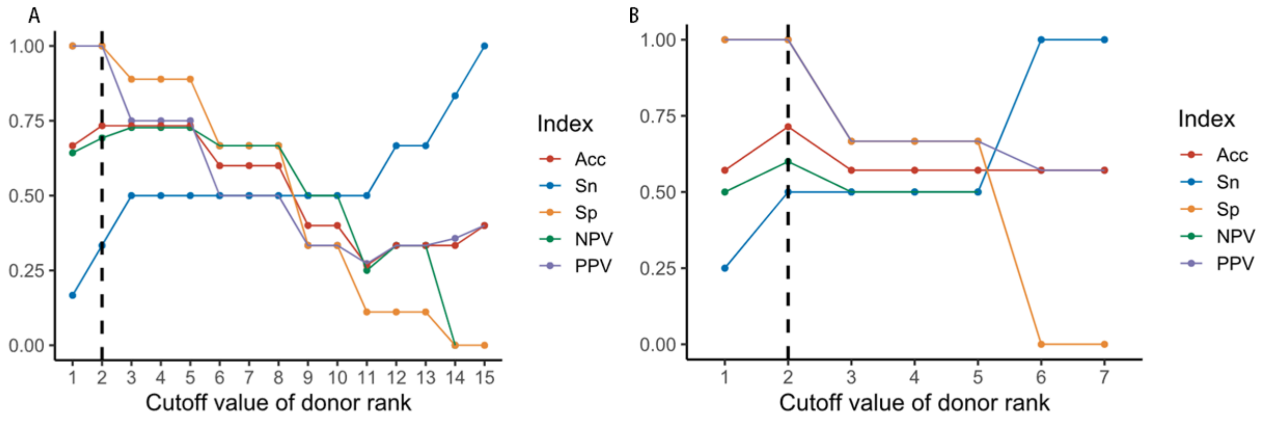

Fig S7. Performances of donor-recipient matching model at different cutoff values of donor ranks, represented by accuracy (Acc), sensitivity (Sn), specificity (Sp), negative predictive value (NPV), and positive predictive value (PPV) in clinical studies of PRJEB33851 (A) and PRJNA438164 (B). The model showed best performance (highest PPV, Sp, and Acc) when the cutoff was set to 2 in both trials, indicating the patients who received FMT from donors recommended by the matching model were all effective, although more trials with large sample size are needed for validation.
